# *TaMIR397-6A* and *-6B* Homoeologs Encode Active miR397 Contributing to the Regulation of Grain Size in Hexaploid Wheat

**DOI:** 10.3390/ijms25147696

**Published:** 2024-07-13

**Authors:** Putong Wang, Yujie Wu, Junhui Zhang, Jiao Si, Xiaoteng Wang, Zhongfa Jiao, Xiaodan Meng, Li Zhang, Fanrong Meng, Yongchun Li

**Affiliations:** 1 Henan Technology Innovation Center of Wheat, State Key Laboratory of Wheat and Maize Crop Science, College of Agronomy, Henan Agricultural University, Zhengzhou 450046, China; 2 College of Life Sciences, Henan Agricultural University, Zhengzhou 450046, China

**Keywords:** microRNA, grain filling, target gene, overexpression, short tandem target mimic

## Abstract

Wheat is one of the most important food crops globally, and understanding the regulation of grain size is crucial for wheat breeding to achieve a higher grain yield. MicroRNAs (miRNAs) play vital roles in plant growth and development. However, the miRNA-mediated mechanism underlying grain size regulation remains largely elusive in wheat. Here, we report the characterization and functional validation of a miRNA, TamiR397a, associated with grain size regulation in wheat. The function of three *TaMIR397* homoeologs was determined through histochemical β-glucuronidase-dependent assay. MiRNA expression was detected using quantitative reverse transcription polymerase chain reaction (qRT-PCR), and the function of TamiR397a was validated through its transgenic overexpression and repression in wheat. It was found that *TaMIR397-6A* and *TaMIR397-6B* encode active TamiR397a. The expression profiling indicated that TamiR397a was differentially expressed in various tissues and gradually up-regulated during grain filling. The inhibition of TamiR397a perturbed grain development, leading to a decrease in grain size and weight. Conversely, the overexpression of TamiR397a resulted in increased grain size and weight by accelerating the grain filling process. Transcriptome analysis revealed that TamiR397a regulates a set of genes involved in hormone response, desiccation tolerance, regulation of cellular senescence, seed dormancy, and seed maturation biological processes, which are important for grain development. Among the down-regulated genes in the grains of the TamiR397a-overexpressing transgenic plants, 11 putative targets of the miRNA were identified. Taken together, our results demonstrate that TamiR397a is a positive regulator of grain size and weight, offering potential targets for breeding wheat with an increased grain yield.

## 1. Introduction

Wheat is one of the most important food crops worldwide, feeding more than 2.5 billion people and providing about 20% of the dietary calories consumed by the global population [1]. The increasing world population and the frequent occurrence of extreme weather conditions due to climate change have posed severe threats to the safe production of wheat and global food security [2]. Therefore, effectively improving the wheat grain yield will always be the focus of crop molecular breeding [3]. Grain weight, a major factor in wheat yield, is determined by the developmental regulation of wheat grains. Hence, it is imperative to improve our understanding of the molecular mechanisms of grain development in wheat. The rapid development and application of molecular technologies, such as genome sequencing, transcriptomics, and proteomic exploration, have greatly advanced the study of the genes, proteins, and regulatory networks involved in regulating wheat grain development [4,5,6]. Some genes associated with the developmental regulation of wheat grains have been characterized. It was reported that the ATP-binding cassette transporter C (ABCC) plays important roles in the developmental regulation of wheat grains [7], and trehalose 6-phosphate (T6P) is involved in the starch biosynthetic regulation of wheat grains [8]. Additionally, genes associated with signal transduction, carbohydrate metabolism, cell division, and proliferation have also been reported to play important roles in the regulation of wheat grain development [9,10]. However, wheat is an allohexaploid crop with a large genome, and the molecular mechanism and how the genes act together in networks to regulate grain development remain to be disentangled [11].

MicroRNAs (miRNAs) are a class of small non-coding RNAs with a length of 20–24 nt which play crucial regulatory roles in many biological processes, including plant growth and development [12]. Many miRNAs are linked to the regulation of grain development in rice. The grain width and thickness of miR5504 mutants were significantly decreased [13], miR159 and miR167 downstream play an important role in the regulation of grain filling and grain size [14], miRNA1432 negatively regulates grain weight by affecting the grain filling rate [15], the overexpression of miR535 increased grain length [16], the suppression of miR396 substantially increased grain size [17], and the overexpression of miR397 increased the grain size and yield [18]. In wheat, some studies have shown that miRNAs play important regulatory roles in wheat kernel development. It was found that some conserved miRNA families (such as miR156, miR169, miR166, miR164, and miR160) and novel miRNAs (such as tae-miR2003a and tae-miR021b) are associated with grain development [19,20]. Members of miR171 and miR396 were highly expressed in developing grains at seven days after anthesis, suggesting their potential roles in the early stages of grain development [21]. Although a large number of miRNAs are differentially expressed during wheat grain development, there has rarely been functional validation of grain-filling-associated miRNAs.

In our previous study, a total of 86 conserved miRNAs were identified as potentially involved in regulating wheat grain filling processes [22]. Particularly, miR397a showed a gradual increase in expression levels during wheat kernel development, suggesting its crucial role in regulating grain development. To further investigate the biological function of miR397a in controlling wheat grain development, this current research focused on characterizing three homoeologous genes of miR397a and confirming its regulatory impact on grain development through genetic manipulation involving both suppression and overexpression of the miRNA. The findings of this study demonstrate that miR397a serves as a positive regulator of grain weight, providing insights into the mechanism of miRNA-mediated grain development regulation and offering new perspectives for molecular breeding strategies aimed at enhancing wheat grain yield.

## 2. Results

### 2.1. Identification of TaMIR397 Homoeologs in Wheat

**Figure 1:**
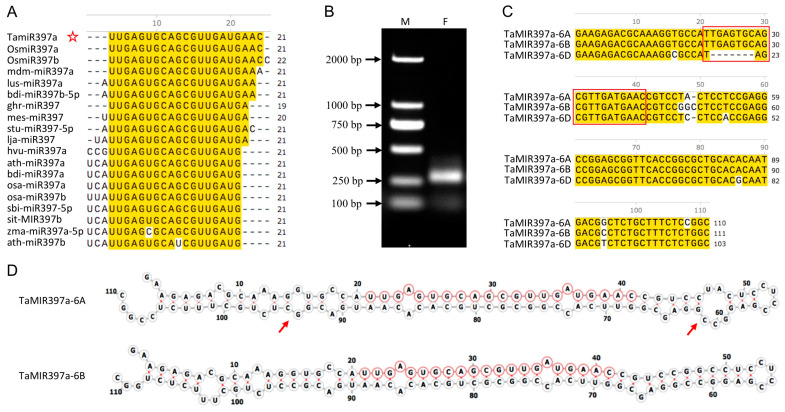
Characterization of TamiR397a and its genomic homoeologs. (**A**) Sequence alignment of miR397 members. TamiR397a is marked with a red five-pointed star. Nucleotides that match those of TamiR397a were highlighted in yellow. Os (*Oryza sativa*), mdm (*Malus domestica*), lus (*Linum usitatissimum*), ghr (*Gossypium hirsutum*), mes (*Manihot esculenta*), stu (*Solanum tuberosum*), lja (*Lotus japonicus*), hvu (*Hordeum vulgare*), ath (*Arabidopsis thaliana*), bdi (*Brachypodium distachyon*), osa (*Oryza sativa*), sbi (*Sorghum bicolor*), sit (*Setaria italica*), zma (*Zea mays*). (**B**) Cloned cDNA fragment of *TaMIR397a*. M, DNA marker DL2000; F, fragment amplified. (**C**) Sequence alignment of three homoeologs. The consensus nucleotides were highlighted in yellow. The region corresponding to mature miRNA is indicated with a red box. (**D**) Stem–loop structures of *TaMIR397a-6A* and *TaMIR397a-6B*. Nucleotides in the mature miRNA region are indicated with red circles, and two additional bubble structures of unpaired sequences are marked with red arrows.

In our previous study, a microRNA (5′-UUGAGUGCAGCGUUGAUGAAC-3′) was identified, which exhibited an up-regulated expression pattern during grain development in wheat [22]. The sequence BLAST (Basic Local Alignment Search Tool) search against the Plant Non-coding RNA Database (PNRD) at http://structuralbiology.cau.edu.cn/PNRD/index.php (accessed on 10 September 2018) revealed that the miRNA belongs to the miR397 family. Its sequence is identical to that of OsmiR397a, leading to its designation as TamiR397a (Figure 1A). The BLASTN search against the wheat genomic sequence (http://plants.ensembl.org/Triticum_aestivum/Tools/Blast (accessed on 13 September 2018 and 16 August 2021), IWGSC RefSeqv2.1) revealed that TamiR397a had three potential transcribing sites in the sixth group of homoeologous chromosomes, designated as *TaMIR397a-6A*, *TaMIR397a-6B*, and *TaMIR397a-6D*, respectively. Three homoeologs of *TaMIR397a* were cloned through cDNA amplification (Figure 1B; Supplementary File S1). Sequence alignment indicated that the precursors of TamiR397a shared 96.4% sequence identity between the A and B subgenomes, and the sequences in the region of mature TamiR397a were identical (Figure 1C). In *TaMIR397a-6D*, seven nucleotides were missing in the region corresponding to the mature TamiR397a, causing it to lose its ability to generate the miRNA. Secondary structural prediction showed that the RNA folding model of *TaMIR397a-6A* was very similar to that of *TaMIR397a-6B*, except for two additional unmatched bubble structures on the left and right sides of the mature miRNA region in *TaMIR397a-6A* (Figure 1D).

**Figure 2:**
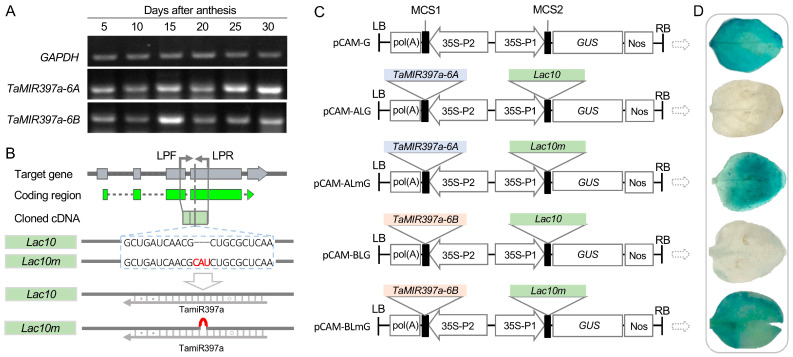
Functional identification of *TaMIR397a-6A* and *TaMIR397a-6B*. (**A**) Expression of *TaMIR397a-6A* and *TaMIR397a-6B*. *GAPDH*, the endogenous control gene encoding glyceraldehyde-3-phosphate dehydrogenase. (**B**) Diagrams of target fragment cloning and mutation. The target gene is TraesCS4A02G096400 encoding a laccase (Lac10). The primers LPF and LPR were used to clone the cDNA fragment of *Lac10* and generate its mutant Lac10m. Red indicates the bulge sequences in the miRNA binding region. (**C**) Diagrams of T-DNA structures. MCS1 and MCS2 indicate two multiple cloning sites. LB and RB represent the left and right borders of T-DNA. *GUS*, β-glucuronidase gene. (**D**) Histochemical GUS assay of tobacco leaves transformed by different T-DNA structures.

The similar stem–loop structure of these two homoeologs raised questions about their ability to produce mature TamiR397a. Considering that TamiR397a is up-regulated during grain filling [22], the expression of *TaMIR397a-6A* and *TaMIR397a-6B* was investigated in developing wheat grains. The results indicated that both homoeologs exhibited up-regulated expression, suggesting that both of them contribute to the production of TamiR397a (Figure 2A). To verify the ability of *TaMIR397a-6A* and *TaMIR397a-6B* to generate TamiR397a, a cDNA fragment of *Lac10* (TraesCS4A02G096400), one of the target genes of TamiR397a, was cloned for the target cleavage assay based on histochemical β-glucuronidase (GUS) analysis (Figure 2B). Additionally, a mutant fragment of *Lac10* (*Lac10m*) was generated, in which three nucleotides were inserted between the 9th and 10th nucleotides of the miRNA binding site. When mRNA of *Lac10m* is paired with TamiR397a, a bulge will form at the cleavage site, causing the inhibition of TamiR397a’s ability to suppress *Lac10m* (Figure 2B). The histochemical GUS assay results showed that the GUS activity was suppressed in pCAM-ALG-transformed tobacco leaves, where *TaMIR397a-6A* was co-expressed with the *Lac10-GUS* fusion gene, indicating that *TaMIR397a-6A* could effectively down-regulate its target gene *Lac10* (Figure 2C,D). In tobacco leaves transformed with pCAM-ALmG, the inhibitory effect of *TaMIR397a-6A* on GUS was eliminated. This suggests that the cleavage site mutation of *Lac10m* effectively blocked the inhibitory function of *TaMIR397a-6A*. Combining the positive and negative test results mentioned above, it is concluded that *TaMIR397a-6A* functions in producing TamiR397a. Similarly, the histochemical GUS assay was performed on *TaMIR397a-6B*, and the results demonstrated that *TaMIR397a-6B* is also a functional gene of TamiR397a. 

### 2.2. TamiR397a Exhibits Differential Expression in Various Tissues

**Figure 3:**
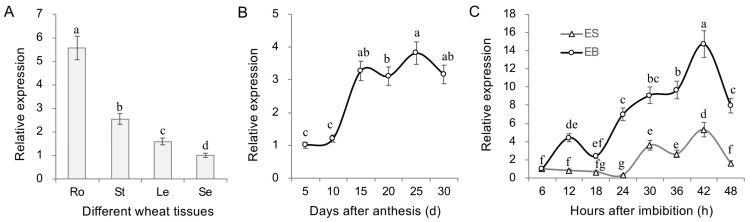
Spatiotemporal expression patterns of TamiR397a in wheat. (**A**) Relative expression of TamiR397a in different tissues. Ro—roots; St—stems; Le—leaves; Se—seeds. (**B**) Expression of TamiR397a in developing wheat grains. (**C**) Expression of TamiR397a during seed germination. EB refers to embryo and bud tissues of germinating seeds, while ES denotes endosperm tissues of germinating wheat seeds. Error bars represent the mean ± SD (*n* = 3) of three biological replicates, and bars or points with the same letters indicate statistically non-significant differences (*p* < 0.05).

To explore the expression characteristics of TamiR397a, the abundance of the miRNA in different tissues was detected. The results showed that the expression level of TamiR397a was relatively higher in the roots and gradually decreased in stems, leaves, and mature seeds (Figure 3A). In developing wheat grains, TamiR397a showed significantly higher expression at the time point of 15–30 days after anthesis (DAA) compared to 5–10 DAA (Figure 3B), suggesting that TamiR397a is involved in the regulation of grain filling. During germination, the expression level of TamiR397a gradually increased before decreasing 48 h after imbibition in embryo and bud tissues (Figure 3C). In contrast, the expression of TamiR397a was relatively lower in endosperm tissues, with only small fluctuations in the expression levels at 30–42 h after imbibition. The differential expression patterns observed during germination suggest that TamiR397a plays a specific role in seed germination.

### 2.3. Inhibition of TamiR397a Perturbs Wheat Kernel Development

**Figure 4:**
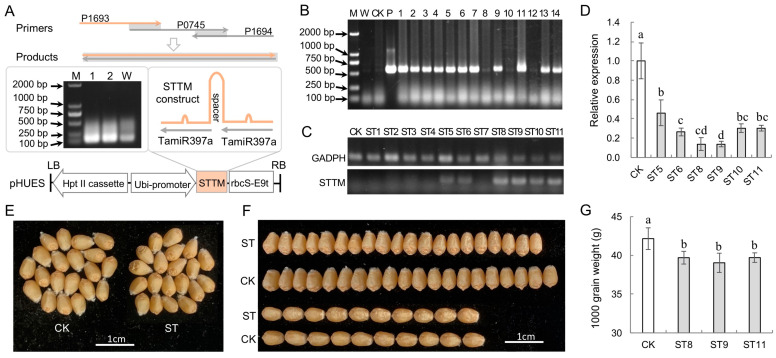
Suppression of TamiR397a by short tandem target mimic (STTM) methods. (**A**) Construction of the ubiquitin promoter-driven *TamiR397a-STTM* for genetic transformation. M, DNA marker DL2000; 1 and 2 indicate the PCR products using P0745 as a template, and W indicates the use of pure water as a template. (**B**) Identification of transgenic wheat plants. W and CK indicate PCR detection using water and genomic DNA of non-transgenic plants as negative controls; P represents plasmid pHUES as the positive control; 1−14 represent putative transgenic wheat plants. (**C**) Expression of *TamiR397a-STTM*. CK refers to non-transgenic wheat plants, while ST1−11 denotes transgenic plants containing the transgene *TamiR397a-STTM*. GAPDH is the housekeeping gene encoding glyceraldehyde-3-phosphate dehydrogenase used as the internal control. STTM refers to the transcriptional detection of *TamiR397a-STTM* constructs. (**D**) Relative abundance detection of TamiR397a. (**E**,**F**) show the grains of Kenong199 (CK) and *TamiR397a-STTM* T_2_ transgenic line (ST). (**G**) Grain weight variation in STTM transgenic wheat. Scale bars: 1 cm. Error bars represent the mean ± standard deviation (*n* = 3) of three biological replicates, and bars labeled with the same letters indicate statistically non-significant differences (*p* < 0.05).

To investigate the function of TamiR397a in wheat, a short tandem target mimic (STTM) method was used to suppress the endogenous TamiR397a (Figure 4A). The STTM construct (126 bp) was generated by PCR-based amplification using three primers: P1693, P1694, and P0745 (Supplementary Table S1). The cloned fragment was then inserted into the cloning site of pHUE0 to obtain the recombinant expression vector pHUES. This vector was used for the genetic transformation of the wheat variety Kenong199. Finally, 14 T_0_ plantlets were generated, and PCR identification showed that 11 plantlets harbored the transgene *TamiR397a-STTM* (Figure 4B). Transcriptional analysis revealed that only six transgenic plantlets exhibited a relatively higher expression level of the STTM structure (Figure 4C). The expression analysis of TamiR397a revealed that the endogenous TamiR397a was significantly down-regulated in these six transgenic plantlets with a relatively higher expression of the STTM structure. The inhibitory effect varied among these plantlets, showing a general negative correlation with the expression level of the STTM structure in the transgenic plantlets (Figure 4D). According to the repression effects of TamiR397a, three transgenic plantlets (ST8, ST9, and ST11) were selected for further phenotypic analysis. In the T_2_ generation, it was found that the grain size of the transgenic lines became slightly smaller than that of the control (Figure 4E), with the grain length and width reduced by approximately one-twentieth (Figure 4F). Analysis of the grain weight revealed that the grain weight of these three transgenic lines (ST8, ST9, and ST11) was decreased by 5.8% to 7.4% (Figure 4G). These results show that the down-regulated expression of TamiR397a reduces grain size and weight, prompting the question of whether this miRNA plays a positive regulatory role in wheat grain development.

### 2.4. Overexpression of TamiR397a Increases the Grain Size and Weight

**Figure 5:**
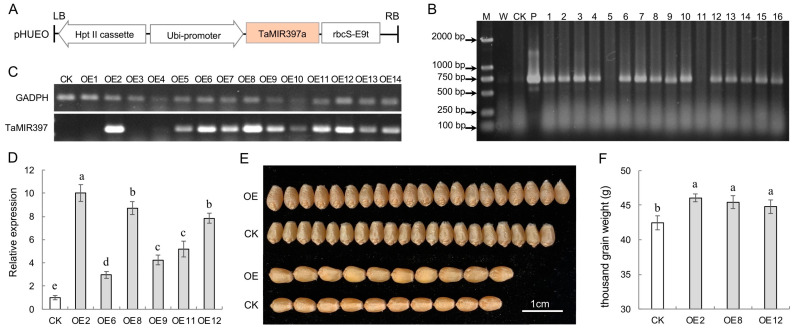
Overexpression of TamiR397a in wheat. (**A**) T-DNA construct for TamiR397a overexpression (OE). (**B**) PCR-based detection of the transgene in putative transgenic plants. W, CK, and P indicate that the PCR template is water, genomic DNA of control (CK) plants, and plasmid of pHUEO, respectively. The numbers 1−16 represent the T_0_ generation wheat plants detected. (**C**) Transcriptional level of the transgene. OE1-14 represents positive transgenic wheat plants. GAPDH refers to the internal control gene that encodes glyceraldehyde-3-phosphate dehydrogenase, while TaMIR397 represents the transgene *TaMIR397a-6B*. (**D**) Relative expression of TamiR397a in CK and transgenic wheat lines with higher expression of the transgene. (**E**) Comparison of grain size between CK and OE. (**F**) Grain weight analysis of CK and OE lines. Error bars represent the mean ± standard deviation (*n* = 3) of three biological replicates, and bars with the same letters indicate statistically non-significant differences (*p* < 0.05).

To verify the effects of TamiR397a overexpression on wheat grain development, the DNA fragment of *TaMIR397a-6B* was cloned using the primer pair P1715 and P1716 (Supplementary Table S1). Subsequently, it was inserted into the cloning site of pHUE0, resulting in the generation of the plant expression vector pHUEO (Figure 5A). After genetic transformation and plantlet regeneration, 16 T_0_ generation plants were obtained, and the transgenic sequence was detected in 14 of them (labeled OE1–14) based on PCR identification of genomic DNA (Figure 5B). The expression level analysis revealed variations in the transgene expression levels among the 14 transgenic plants. The transgene was highly expressed in OE2, OE6, OE8, OE9, OE11, and OE12, relatively lower in OE5, OE7, OE10, OE13, and OE14, and much lower in OE1, OE3, and OE4 (Figure 5C). The abundance detection of TamiR397a showed that the expression level of this miRNA in OE2, OE8, and OE12 was significantly increased, reaching 7.8–10.0 times that of the control (CK) (Figure 5D). Further analysis of the grain traits of these three overexpressed (OE) lines demonstrated an obvious increase in grain size. The length and width of the transgenic wheat grains increased by approximately 7.1% and 5.8% (Figure 5E). Additionally, the grain weight of OE2, OE8, and OE12 increased by 8.5%, 7.1%, and 5.5%, respectively (Figure 5F).

### 2.5. TamiR397a Is Involved in the Regulation of Grain Filling

**Figure 6:**
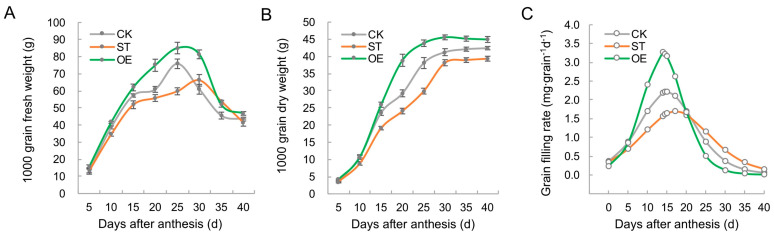
Characterization of grain weight changes during grain development. The changes in fresh (**A**) and dry (**B**) weight of grains. (**C**) Patterns of grain filling rate. CK, control; ST, TamiR397a-STTM; OE, TamiR397a overexpression. Error bars represent the mean ± standard deviation (*n* = 3) of three biological replicates.

To investigate the regulatory role of TamiR397a in grain formation, the characteristics of the wheat grain filling process were analyzed. The results showed that the fresh weight (Figure 6A) and dry weight (Figure 6B) of wheat grains overexpressing TamiR397a (OE) were higher than those of the control (CK), especially during the period of 15–30 days after anthesis (DAA). In contrast, under the inhibition of TamiR397a expression, the fresh weight of the TamiR397a-STTM (ST) grains was lower than that of the CK before 25 DAA and higher between 30 and 35 DAA (Figure 6A). The higher fresh weight of the ST grains at the later stage of development may be related to the slowing down of the grain development process caused by TamiR397a repression. The dry weight analysis revealed that the accumulation of dry matter in the ST grains was significantly lower than that in the CK grains (Figure 6B). Considering that the fresh weight of the ST grains at the late stage of grain formation was higher than that of the CK, the dehydration rate of the ST grains was slower. The analysis of the grain filling rate showed that the maximum filling rate of the OE grains was the highest, while that of the ST grains was the lowest (Figure 6C). Moreover, the overexpression of TamiR397a accelerated the grain filling process, reaching the maximum filling rate at 14.0 DAA, while the CK and ST grains reached the maximum filling rate at 15.1 and 17.3 DAA, respectively. Based on the above results, it was found that TamiR397a plays a positive regulatory role during grain filling in wheat.

### 2.6. Differentially Expressed Genes in the OE and ST Wheat Grains

**Figure 7:**
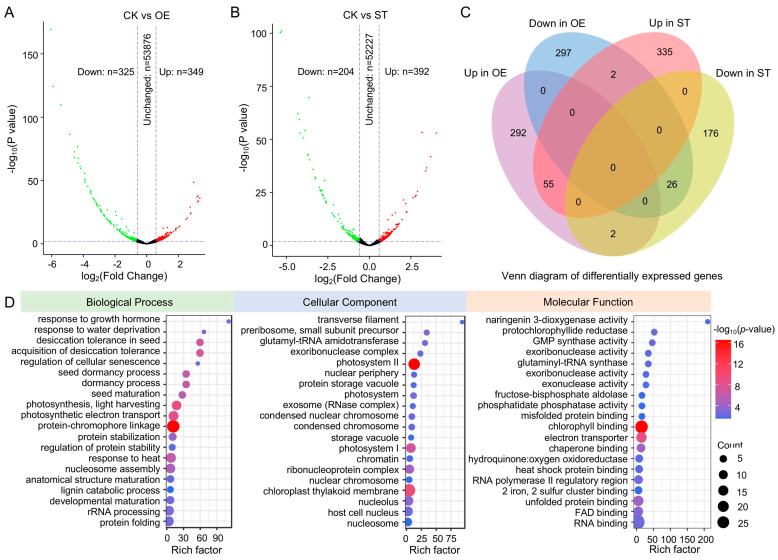
Genes regulated by TamiR397a in wheat grains. (**A**,**B**) present volcano plots of differentially expressed genes (DEGs) in OE (TamiR397a overexpression) and ST (TamiR397a-STTM) wheat grains compared to CK (control), respectively. The vertical dashed lines indicate the log_2_(fold change) ratios of −0.58 and +0.58, while the horizontal dashed lines indicate −log_10_ (*p* value) of 2. Red dots and green dots represent significantly up- and down-regulated genes, respectively. Black dots represent genes that have not changed. (**C**) Distribution of up- and down-regulated genes in OE and ST compared to CK. (**D**) Gene Ontology terms of putative genes regulated by TamiR397a in OE or ST grains.

To understand the gene network regulated by TamiR397a in wheat grains, the transcriptomes of the OE, ST, and CK grains were analyzed using RNA sequencing (RNA-seq). The transcriptome comparison revealed 349 up-regulated and 325 down-regulated genes in the OE grains compared to the CK (Figure 7A; Supplementary Table S2) and 392 up-regulated and 204 down-regulated genes in the ST grains (Figure 7B; Supplementary Table S3). Among these differentially expressed genes (DEGs), 81 DEGs (55 up-regulated and 26 down-regulated) were found to be shared in both the OE and ST kernels (Figure 7C). This suggests that these genes may not be regulated by TamiR397a in wheat grains. The remaining DEGs, excluding the 81 DEGs mentioned above, should be considered candidates regulated by miR397a (CRm). The analysis of the Gene Ontology (GO) terms enriched in these CRm genes revealed the statistical importance of growth hormone, desiccation tolerance, regulation of cellular senescence, seed dormancy process, and seed maturation for biological process terms (Figure 7D). Accordingly, these enriched biological processes are very important to grain filling and seed maturation, suggesting that TamiR397a plays key roles in the developmental regulation of wheat grains. The enriched biological processes terms also include photosynthesis, regulation of protein stability, and lignin catabolic pathways. In terms of cellular components, pathways related to photosystems and chloroplasts were enriched, which are essential for grain filling and seed formation. The enriched molecular function terms included those involved in the photosynthetic pathway (such as chlorophyll binding and cyclic electron transporter), protein binding, DNA binding, RNA binding, and FAD binding.

### 2.7. Identification of TamiR397a Targets in Wheat Grains

In plants, miRNAs are involved in the molecular regulation of development by post-transcriptionally inhibiting their target genes. To explore the targets regulated by TamiR397a in wheat grains, target prediction was conducted using down-regulated genes in the grains of the TamiR397a-overexpressed transgenic plants, resulting in the identification of a total of 11 candidates (Table 1). The molecular functions of these target-encoded proteins include protein binding (TraesCS5B03G0937500, TraesCS5B03G0709900), nucleic acid binding (TraesCS2B03G0934700, TraesCS4B03G0327300), transmembrane transporting (TraesCS2D03G0440600LC, TraesCS4B03G0965700), catalytic activity (TraesCS3A03G0831400, TraesCS7B03G0395000, TraesCS1B03G0669000), and microtubule binding (TraesCS3B03G0720100). NewGene_945 is a gene with an unknown function that may encode for a long non-coding RNA (Supplementary File S2).

**Figure 8:**
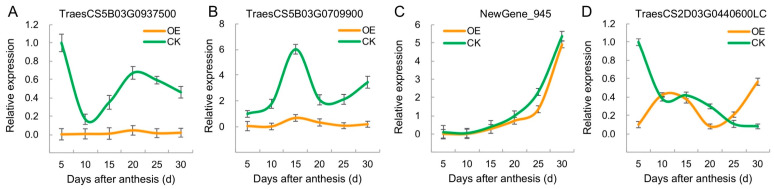
Expression of potential targets of TamiR397a in developing wheat grains. (**A**) Expression patterns of the TraesCS5B03G0937500 gene, which encodes a protein containing a tetratricopeptide repeat (TPR) domain, in OE (TamiR397a overexpression) and CK (control) grains. (**B**) Expression profiles of the TraesCS5B03G0709900 gene (encoding an F-box domain-containing protein) in OE and CK grains. (**C**) Expression patterns of NewGene_945 (encoding a long non-coding RNA) in OE and CK grains. (**D**) Expression patterns of the TraesCS2D03G0440600LC gene (encoding a sugar transporter protein) in OE and CK grains.

Further expression analysis of the four candidate targets revealed that the identified genes were repressed to varying degrees during the development of the OE grains (Figure 8A–D). For TraesCS5B03G0937500, it was down-regulated before 10 DAA, then gradually up-regulated, and reached the highest expression level at 20 DAA, and this was followed by a slight decrease in the CK grains (Figure 8A). For TraesCS5B03G0709900, a single peak of up-regulated expression was observed, with the highest expression level occurring at 15 DAA for the CK (Figure 8B). In the CK, the expression patterns of these two genes were associated with the grain filling process, while in the OE grains, these two genes were significantly suppressed during grain development. This suggests that these two genes play a role in regulating grain filling. NewGene_945 (NG945) was gradually up-regulated during the development of the wheat grains. The overexpression of TamiR397a only exhibited a slight inhibitory effect on its expression at 20 DAA in the OE grains (Figure 8C). This suggests that this gene is not implicated in the TamiR397a-mediated regulation of grain filling. TraesCS2D03G0440600LC was down-regulated during grain development in the CK, and significant inhibition was only detected at 5 DAA and 20 DAA in the OE grains (Figure 8D), indicating that this gene can be regulated by multiple pathways besides TamiR397a-mediated repression.

## 3. Discussion

The development process of wheat grains directly determines the final wheat yield. Therefore, it is of great importance to explore the molecular regulation mechanism of grain development for molecular breeding focused on achieving a higher grain yield in wheat [11]. Generally, the developmental process of wheat grain involves three successive stages: cellularization, grain filling, and maturation/desiccation [23]. The first stage lasts until up to 10 DAA and involves cell division and differentiation, which mainly determine the sink capacity [24]. The grain filling stage is characterized by the onset of the accumulation of storage materials, including starch and gluten proteins, and lasts up to 20 days (i.e., from 11 to 30 DAA) [23]. During the initial stage of grain filling (11–16 DAA), the endosperm cells continue to divide and create storage compartments [25]. After 16 DAA, endosperm cell division ceases, and protein and starch granules begin to accumulate rapidly [26]. In this study, it was found that the expression level of TamiR397a increased significantly after 15 DAA and maintained a high expression level during the grain filling stage. This suggests that TamiR397a is involved in the regulation of grain filling. Some previous studies have also identified a set of miRNAs that are differentially expressed in developing wheat grains. It is believed that miRNAs play critical regulatory roles in wheat grain development [19,20,22]. However, little is known about the mechanisms of miRNA-mediated regulation of wheat grain development, and functional validation of miRNAs associated with wheat grain development has rarely been conducted [11]. In this study, the regulatory function of TamiR397a was investigated, and it was confirmed that TamiR397a positively regulates grain development by accelerating the grain filling process, resulting in an increased grain size and weight. 

MiRNAs play important regulatory roles in plant growth and development by repressing their target genes at the post-transcriptional level [11,12,13]. To identify the genes regulated by TamiR397a in wheat grains, differentially expressed genes in transgenic OE and ST wheat plants were analyzed in this study. A set of genes potentially regulated by TamiR397a was identified. This includes genes involved in hormone response, desiccation tolerance, regulation of cellular senescence, seed dormancy, and seed maturation biological processes, which are crucial for grain development and yield formation in wheat. Interestingly, two genes (TraesCS5B03G1114400LC and TraesCS1B03G0418900) were found to be up-regulated in one condition (OE) and down-regulated in another (ST), while the other two genes (TraesCSU03G0095600LC and TraesCSU03G0095700LC) exhibited the opposite pattern (Figure 7C). These genes are likely influenced by TamiR397a indirectly. Functional analysis revealed that TraesCS5B03G1114400LC encodes a protein similar to 6-phosphofructo-2-kinase, TraesCS1B03G0418900 encodes a ubiquitin carboxyl-terminal hydrolase, and TraesCSU03G0095600LC and TraesCSU03G0095700LC encode proteins with unknown functions. The expression levels of TraesCS5B03G1114400LC and TraesCS1B03G0418900 were relatively low during grain filling (Supplementary Files S3A and S3B), so their up-regulation in the OE wheat grains may be positively associated with grain size and weight. TraesCSU03G0095600LC was highly expressed at the time point of 30 days after anthesis (Supplementary File S3C), indicating its correlation with grain maturation. No transcript of TraesCSU03G0095700LC was detected in the WheatExp database. Recently, it was reported that both 6-phosphofructo-2-kinase and ubiquitin carboxyl-terminal hydrolase are involved in the regulation of grain development. Mutation of the 6-phosphofructo-2-kinase gene disrupted glycolysis and energy metabolism, affecting the synthesis of grain storage compounds in rice [27]. The ubiquitin-specific protease OsUBP15 acts as a positive regulator of rice grain size [28,29]. Therefore, the functional verification of TraesCS5B03G1114400LC and TraesCS1B03G0418900 will be an intriguing topic in our upcoming research on grain size regulation in wheat.

Among the genes repressed by TamiR397a in the OE wheat grains, 11 target genes were predicted, including TraesCS5B03G0937500 and TraesCS5B03G0709900. Functional annotation indicated that TraesCS5B03G0937500 encodes an uncharacterized protein containing a typical tetratricopeptide repeat (TPR) domain (TaTPR), which was highly homologous to *Arabidopsis* TPR-like superfamily protein (AT3G26580) and rice TPR-containing protein (BGIOSGA012921), with amino acid sequence identities of 59.6% and 77.4%, respectively. It has been found that AT3G26580 is involved in leaf development [30], while the function of BGIOSGA012921 remains unclear. Generally, TPR domains and their scaffold complexes are involved in a wide range of molecular regulation, including the assembly of multiprotein complexes, cell cycle regulation, transcriptional control, and protein folding [31]. In *Arabidopsis*, TPR-containing proteins have been found to be essential for phytohormone responses, root development, plastid distribution, and photosynthetic machinery [32]. Recently, a TPR-containing protein called FLOURY ENDOSPERM 2 was found to be involved in the regulation of storage compound accumulation, which is associated with seed size and quality in *Arabidopsis* [33] and rice [34]. Therefore, it is speculated that the TaTPR identified in this study may play an important role in wheat grain development and grain filling regulation.

For another candidate target gene, TraesCS5B03G0709900, it encodes an F-box domain-containing protein (TaFbox). The F-box proteins are substrate recognition components of the SCF (Skp1, Cullin, and F-box) E3 ubiquitin ligase complex [35]. By selectively targeting the regulatory proteins for ubiquitination and 26S proteasome-mediated degradation. Plant growth and development are regulated by a variety of proteolytic pathways, among which the F-box-mediated proteolytic pathway plays an important role in determining the fate of key proteins involved in the developmental regulation of plants [36]. It has been found that F-box proteins participate in various developmental processes, including cell division, development, and plant hormone responses [37]. The F-box proteins constitute a superfamily. At least 897, 971, and 1796 F-box members have been identified in *Arabidopsis*, rice, and wheat, respectively [35,38,39]. Despite their importance and large number of members, the molecular functions of F-box proteins remain largely unknown in plants. Recently, an F-box protein, FBX206, was identified. It acts as a negative regulator in brassinosteroid signaling and controls grain size and yield in rice [36]. Usually, F-box proteins contain highly variable protein–protein interaction domains at the carboxyl-terminal regions, such as Leucine-rich repeats (LRRs), WD-40, Armadillo (Arm), TPRs, and others, which serve to specifically recruit substrate proteins for ubiquitination and subsequent degradation [40]. In this study, the newly identified target gene TraesCS5B03G0709900 encodes a TaFbox protein consisting of an F-box and an LRR domain. However, the current knowledge of this type of LRR-containing F-box protein is still very limited [35]. Further, it may be an interesting research direction to explore the molecular function of the TaTPR and TaFbox coding genes in wheat grain development and grain yield formation.

## 4. Materials and Methods

### 4.1. Wheat Planting and Sampling

The wheat variety ‘Kenong 199’ was selected for this study and planted in an artificial climate chamber with 16 h of light and 8 h of darkness at 24 °C. Tissue samples used to analyze the spatiotemporal expression patterns included roots at the three-leaf stage, stems, and leaves at 20 days after anthesis, and mature seeds. Developing wheat grains were collected at 5, 10, 15, 20, 25, and 30 days after anthesis to detect the expression levels of miR397a and candidate genes. During seed germination, the embryo (including germinated buds) and endosperm tissues were isolated at 6, 12, 18, 24, 30, 36, 42, and 48 h after imbibition, respectively. The experimental samples included three biological replicates. All samples were rapidly frozen using liquid nitrogen and then stored in a −80 °C freezer.

### 4.2. Gene Cloning and Functional Identification of TaMIR397a

The DNA fragments of the *TaMIR397a* homoeologs were amplified by polymerase chain reaction (PCR) using primers P1674 and P1675 (Supplementary File S4A). The PCR product was purified, ligated with the pMD19-T vector, and then transformed into competent *Escherichia coli* (*E. coli*) cells. To distinguish the three homoeologs of *TaMIR397a*, recombinant *E. coli* colonies were identified by PCR (Supplementary File S4B) using common primers (P0085 and P0086 located in the T-vector region) and *TaMIR397a-6A*, *-6B*, and *-6D* specific primers (P1706–P1708) and confirmed by Sanger sequencing. All the primers are listed in Supplementary Table S1.

To verify whether the TaMIR397a genes can produce mature TamiR397a, an identification system relying on histochemical β-glucuronidase (GUS) analysis was used. Firstly, a fragment of *TaMIR397a-6A* or *TaMIR397a-6B* was amplified using the primer pair P2141/P2142 (Supplementary File S4C) and inserted into the *Xho*I-*Apa*I site of the pCAM-G vector. Secondly, a target gene fragment of the *Lac10* or *Lac10* mutation (*Lac10m*) was fused downstream of the GUS gene in pCAM-G. The *Lac10* gene (TraesCS4A02G096400) was amplified using the LPF and LPR primers (Supplementary File S4D). *Lac10m* was generated through PCR amplification (Supplementary File S4E): (1) The *Lac10* and *Lac10m* fragments were amplified using the primer pairs LPF/P2330 and LPR/2329, respectively. (2) A mixture of the two fragments produced in step (1) served as a template, and the mutant fragment *Lac10m* was generated by PCR using the primers LPF and LPR.

GUS activity was detected through histochemical staining with 5-bromo-4-chloro-3-indolyl-β-D-GlcA. Initially, the recombinant *Agrobacterium tumefaciens* strain EHA105 was transformed with individual vectors. Solutions of recombinant *Agrobacterium* (0.5 OD600) were then infiltrated into the leaves of *N. benthamiana*. Treated tobacco seedlings were grown under dark conditions for three days. Subsequently, leaves infiltrated with *Agrobacteria* were isolated and incubated overnight in a staining buffer containing 1 mM 5-bromo-4-chloro-3-indolyl-β-D-GlcA, 100 mM sodium phosphate (pH = 7), 10 mM EDTA, 0.5 mM K_4_Fe(CN)_6_, 0.5 mM K_3_Fe(CN)_6_, and 0.1% (*v*/*v*) Triton X-100 at 37 °C. Finally, the leaves were rinsed in 70% ethanol and washed several times in 95% ethanol to remove the chlorophyll.

### 4.3. Bioinformatics Analysis

MicroRNA and DNA sequence alignments were performed using SnapGene (version 4.2.11). The mature miRNA sequences were analyzed using BLASTN in the Plant Non-coding RNA Database (http://structuralbiology.cau.edu.cn/PNRD/ (accessed on 10 September 2018)). Genomic searches for TaMIR397a genes and the target genes of TamiR397a were conducted in the wheat genome database on Ensembl Plants (http://plants.ensembl.org/index.html (accessed on 13 September 2018 and 15 August 2023)). Prediction of the TamiR397a targets was conducted using psRNATarget (https://www.zhaolab.org/psRNATarget/ (accessed on 25 September 2018 and 16 October 2021)). The stem–loop structures of TaMIR397a were predicted using the RNAfold program available at http://rna.tbi.univie.ac.at/cgi-bin/RNAWebSuite/RNAfold.cgi (accessed on 19 September 2018). Volcano plots were generated using the volcano plotting tools on the BMKCloud platform (www.biocloud.net (accessed on 28 September 2021)). 

### 4.4. Quantitative Expression Analysis

The expression of TamiR397a was detected using the *TransScript*^®^ Green miRNA Two-Step qRT-PCR SuperMix kit (Code # AQ202-01, TranGen Biotech, Beijing, China). The specific primer P0929 was used along with the primer pair P1303/P1304 to detect the non-coding RNA U6 (GB#: X63066) as the endogenous control. The expression patterns of TraesCS5B03G03937500 (*TPR*), TraesCS5B03G0709900 (*F-box/LRR*), TraesCS2D03G0440600LC (*STP*), and NewGene_945 were identified using quantitative real-time PCR (qRT-PCR) with specific primer pairs (Supplementary File S4F), P3184/P3188, P3192/P3194, P3198/P3200, and P3204/P3205 (Supplementary Table S1), respectively. The *GAPDH* (GenBank #: EF592180) gene was used as an endogenous control. The total RNA was isolated using the TransZol Plant kit (#ET121-01, TransGen Biotech, Beijing, China), and the RNA integrity was assessed based on the RNA Integrity Number (RIN) value (>8) and the 28S/18S ratio (>1.8). The qRT-PCR reaction system (20 μL) included SYBR Green I PCR Master Mix (10 μL), 10 μM of forward and reverse primers (1 μL of each), cDNA template (2 μL), and ddH_2_O (6 μL). The expression levels of TamiR397a and the candidate genes were calculated using the 2^−∆∆CT^ method.

### 4.5. Genetic Transformation and Identification of the Transgenic Plants

For the overexpression of TamiR397a, the genomic DNA sequences of *TaMIR397a-6B* (253 bp) were amplified using the primer pair P1715/P1716 and cloned into the *Kpn*I site of pHUE0. To inhibit TamiR397a, a short tandem target mimic (STTM) structure for miR397a was created by amplifying a fragment using the primers P1693, P1694, and P0745. Subsequently, the amplified fragment was cloned into the *Kpn*I site of pHUE0, resulting in the generation of the recombinant expression vector pHUES. The genetic transformation was performed by Genovo Biotechnology Co., Ltd. (Tianjin, China). Genomic DNA and total RNA were extracted from the leaves of regenerated transgenic plantlets and corresponding non-transgenic wheat plants (CK) using the CTAB and Trizol methods, respectively. The primer pair P1635/P1696 was used to detect the overexpression cassettes of *TaMIR397a-6B* (686 bp) and *STTM-miR397a* (555 bp) in the genomic DNA. The specific primer pairs P2009/P1677 and P2009/P1733 (Supplementary Table S1) were used to detect the expression of transgenic *TaMIR397a-6B* and *STTM-miR397a*, respectively. The *GAPDH* (GenBank #: EF592180) gene was used as an endogenous control.

### 4.6. Characterization of Wheat Grain Filling

The logistic equation was used to characterize the grain filling process, as reported in previous studies [41,42]. The grain weight (mg) is calculated as follows: W = A/(1 + B × e^−k·t^), and the grain filling rate (G) is evaluated as the derivative of the grain weight: G = (A × k × B × e^−k·t^)/(1 + B × e^−k·t^)^2^. A represents the theoretical maximum grain weight (mg), t represents days after anthesis (d), and B and k are regression coefficients.

### 4.7. Transcriptome and Target Gene Analysis

The OE and ST transgenic wheat grains were collected at 20 days after anthesis (DAA) for transcriptome analysis. RNA sequencing was performed on the Illumina platform by Biomarker Technologies in Beijing, China. Sequencing libraries were prepared using the NEBNext Ultra^TM^ RNA Library Prep Kit for Illumina (NEB, Ipswich, MA, USA), with unique index codes assigned to each sample for sequence attribution. Subsequently, the libraries were sequenced on the Illumina platform, and the raw reads were processed using the BMKCloud online platform (www.biocloud.net (accessed on 13 September 2021)). Raw reads in FASTQ format were subjected to processing via in-house Perl scripts to filter out reads containing adapters or poly-N sequences, as well as low-quality reads. Quality control metrics such as Q20, Q30, GC-content, and the sequence duplication levels were assessed for the resulting clean reads. High-quality clean data were then mapped to the reference genome sequence (IWGSC CS RefSeq v2.1) using the Hisat2 software (HISAT 2.2.1). Gene function annotation was performed by referencing various databases, including NCBI non-redundant protein sequences, NCBI non-redundant nucleotide sequences, Pfam (Protein family), Clusters of Orthologous Groups of proteins, Swiss-Prot, KEGG Ortholog database, and Gene Ontology. The gene expression levels were calculated using the FPKM (fragments per kilobase of transcript per million mapped reads) method. The thresholds for screening differentially expressed genes were set at a *p* value < 0.01 and a fold change > 1.5. Bioinformatics analysis of the differentially expressed genes was performed using BMKCloud (www.biocloud.net (accessed on 24 September 2021)).

### 4.8. Statistical Analysis

Three biological replicates were used in all experiments. IBM SPSS Statistics 22 was used for the statistical analysis. Significant differences were analyzed using one-way analysis of variance (Tukey’s test, *p* < 0.05).

## 5. Conclusions

In summary, we characterized a conserved miRNA, TamiR397a, which is up-regulated during the development of wheat grains. Three homoeologs of *TaMIR397a* were identified in the wheat genome, and only *TaMIR397-6A* and *TaMIR397-6B* could produce active TamiR397a. Suppression of TamiR397a decreased the grain size and weight, while its overexpression accelerated the grain filling and increased the grain size and weight. Eleven putative targets of TamiR397a were identified. Two of them encode a TPR-domain-containing protein and an F-box domain-containing protein, which may play a crucial role in the developmental regulation of wheat grains. Our results lay the foundation for understanding the miRNA-mediated regulation of grain development and provide a potential approach to molecular breeding to improve the grain yield in wheat.

## Figures and Tables

**Table 1:** Putative targets of TamiR397a down-regulated in OE wheat grains.

Gene ID	Description	Fold Change	Target Aligned Fragment
TraesCS5B03G0937500	TPR-domain-containing protein	−10.5	GUUCUUGGAGGCUGCACUCAC
TraesCS5B03G0709900	F-box domain-containing protein	−3.2	ACUUAACAAUGCUGCAUCCAC
NewGene_945	Putative long non-coding RNA	−2.0	CUUCA-CAAUUCUGCAUUCAG
TraesCS2B03G0934700	ATP-dependent DNA helicase	−1.8	CGUCAUCAGCGAUGUACUCUU
TraesCS2D03G0440600LC	Monosaccharide transporter	−1.7	CCUCGUCGGCGCCGCGCUCAA
TraesCS3A03G0831400	TaLaccase3	−1.7	GUUCAUCAACUCUGCGCUCAA
TraesCS4B03G0327300	ATPase family	−1.6	ACUUCUCAACGCUGUACGCGC
TraesCS7B03G0395000	Lysine-specific demethylase JMJ30	−1.6	AGACCUCGGCGAUGCGCUCAA
TraesCS3B03G0720100	Kinesin-like protein KIN-6	−1.6	UGCCAGUGACGCUGCAUUAAA
TraesCS1B03G0669000	Aminotransferase classes I and II	−1.6	GCUCGUGGACGCCGCGCUCGG
TraesCS4B03G0965700	ATPase 11, plasma membrane-type	−1.5	GAUCAUCAGCGCUGUUCUAAC

## Data Availability

All the data from this study are included in the article and Supplementary Materials. For further inquiries, please contact the corresponding author.

## Supplementary Materials

The supporting information can be downloaded at https://www.mdpi.com/article/10.3390/ijms25147696/s1.
