# Supplementary material for: TaMIR397-6A and -6B Homoeologs Encode Active miR397 Contributing to the Regulation of Grain Size in Hexaploid Wheat
Source: Int J Mol Sci. 2024 Jul 13;25(14):7696. doi: 10.3390/ijms25147696 (PMC11276883; doi:10.3390/ijms25147696)
Supplement: Supplementary file 1 [file ijms-25-07696-s001.zip › Supplementary file S1.pdf]

Supplementary File S1: DNA sequences of three homoeologs of *TaMIR397a*

> *TaMIR397a-6A*

AGCCAGATGCAACCAGACACTACTACTGGGGAAGACGAAGAGGAGGAAGAGACGCAAAGGTGCC
ATTGAGTGCAGCGTTGATGAACCGTCCTACTCCTCCGAGGCCGGAGCGGTTCACCGGCGCTGCACA
CAATGACGGCTCTGCTTTCTCCGGCGAAACAGCAAAGCAAGCCTTTTGTTACTAATCCCGACACCA
TTGACACAGGCATTCATCAGCTACTTATATATGCTCATGTGCCGGTGGTGACATGAAGCGGAGAGA
AGGAGCTCAGTTGGGGCCCCAGCAGACGAGGTGGTGCGCCTTGCGCAGCTTCGCG

> *TaMIR397a-6B*

AGCCAGATGCAACGAGATACTACTACTGGGGAAGACGAAGAGGAGGAAGAGACGCAAAGGTGCC
ATTGAGTGCAGCGTTGATGAACCGTCCGGCCTCCTCCGAGGCCGGAGCGGTTCACCGGCGCTGCA
CACAATGACGCCTCTGCTTTCTCTGGCGAAACAATCACCATTGGCACAGGCATTCATCAGCTACCT
GTGCACGCTCATGTGCCGACGGAGGCATGAAGCGGAGAAGAGGAGCTCAGTTGGGGCCCCAGCA
GACGAGGTGGTGCGCCTTTCGCAGCTTCGCG

> *TaMIR397a-6D*

AGCCAGATGCAACGAGATACTACTAGTGGGCAAGACGAAGAGGAGGAAGAGACGCAAAGGCGCC
ATAGCGTTGATGAACCGTCCTCCTCCACCGAGGCCGGAGCGGTTCACCGGCGCTGCACGCAATGA
CGTCTCTGCTTTCTCTGGCGAAACAATCACCATTGACACAGGCATTCATCAGGTATGCTCATGTGC
CGATGGAGACATGAAGCGGAGAAGAGGAGCTCAGTTGGGCCCCCAGCAGACGAGGTGGTGCGCC
TTGCGCAGCTTCGCG
