# Supplementary material for: TaMIR397-6A and -6B Homoeologs Encode Active miR397 Contributing to the Regulation of Grain Size in Hexaploid Wheat
Source: Int J Mol Sci. 2024 Jul 13;25(14):7696. doi: 10.3390/ijms25147696 (PMC11276883; doi:10.3390/ijms25147696)
Supplement: Supplementary file 1 [file ijms-25-07696-s001.zip › Supplementary file S2.pdf]

Supplementary File S2: The sequence annotation of NewGene_945

Sequence alignment of NewGene_945 and long non-coding RNA (TAES_LNC009446.1)

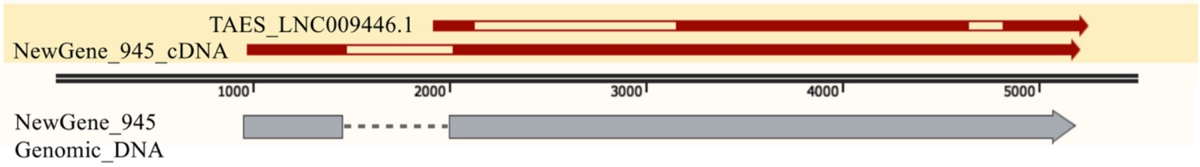

> NewGene_945_cDNA

CTTCTCGAACCACCTCTCCTCTACGGGTCTCCAGGGTTGCGGAAAAAAAAAGCGGCGCCAGCAGGCTACGGGGAGGAGGGC
AGCGCCCTGCCGACGATTTATCTGGCTACTTCCTCCCCGTCGATCCCTGCCTGGACCATACCGTCTCCATCTTGAAGAAGGCT
CGATCTTTTCCGGCGTCAGGTGAGCAGCTGGGTGTCCTTTTTTTCCTGCGGTTTATGGCCGGCGGTGGCCTGAATCTAAGTGG
TGGTTGCCGTCCGGACCGGCCGCCTTCTTTTTGTTCTTCTCCTTGCCTTCCTTGTCACCTCTCCCAATCGCAAGTGCACGCCTC
CACGAGAGCAGGAACAGAGCAGGCATTCATGGAGCAGATTCCTTTTTTTTCTCCACTCCTCTTGCTCCCTTCTGATCGAGCTC
CGCGTAGAGGAGGGGCTCGAGCCAGGTGATGGCCAACACTCCGCCACGGAGGCCAACATGAGGAGGTGCCCAATCTCGCTG
TTGCTCCCCTTTGCGATTGATGAGTTCGTGATGTACGAAAAGAGAATGCCTTAGAAAGACCGGTCATACGAAAGAGCACTTA
TTACTATTGAAATATTATGGTGACGATGTTTAGCAGTCATAAAAGGTATATCTTTTCTCAGATTCATATGGGCTGCCCTTCAA
AGATAAATGTAGAAACAAAGTAGGGAAGATTGTAAGATTGTCCATGGTATTACTTTAATGTGGAGAAATATCACATGCAAAT
TTAGCAATCCGTAATTGTAGTTTTTTACTGGAATGTTTATTTCATAGAATTACAAATTATCATGTTACTTCTACTTCCTTATGG
TATTATACTGTGCCTTATCTCATGACCTAGGGACATTGGTGTCAAGTTTCACATACTTGAACTGTTTAATTGATTGCTTCCATA
AGATCTTCTCCTCAACGTTCCTATGTGTGATGATGTAATCCAGATCAGGCTTTGAGCTGTACTTTTTCCCTCAGCAATACTTTC
AGTGAGAAGTAGCAACACTAATCCATCAATATAGATTATGCGAAGTATTTAACATTGAGGCTGCGTTCGTTATGCAGGGAGA
GAAAATGAAGGTTAAAAATACTTCAAAGGAATAGGACTGAAGGCTACCATAGAACCTACAGGGTCTAGGAACACAGGAATA
AGAGAAAGTAGACTGTTCGGATCACAGCAGCCTTAAGGGTTTTTAAATTGGCCACAAGGCAGCTTTGTCTCTATCTCTCACCC
CATCACGTCACACATCAGCTCATTTTAATTAGCTCTGCATGCTGACTGCCACGATCAGAGCACCTCGTCTCGGTCTTCCGCGA
AAAACATGAGCTCCGGGCAGATTTTTCAATTCCTGTGAAATCGGCATCGAAAGGACAGGTTTCCTTTGGATCACTGTAGGTC
ATTCCTGCGTTTCGAACACCACAAAGGAAAATAAACCTTCATGAACAGTATTCCTTCATGATTCCTGCAAAAATCCTATGAAC
CGAACGGAGCCTGAATATGGTCCAGTTAGTGGCGTATTATTCCTTGCATTCAACACGTGTGTATAACACCTCGACGATGTGTT
ATTCTATATTGAAGTTTTAATGAACCTGAACTCTTCTGATTATTTGTGTAAGCATGAACTCTTGGCGTGCAGGTGTAAGTTG
ATTTTTTTTCAAAAAAAAGGTGCAAGTCCTATCAGGCACATGGACAACTAACATCATGCTTCCTCTGTTTAGACATTACTCT
ACCACGTAGTTGGTCTTTGGTTCTCTAGGAGATCGTGGTCCATATTTGATTAACTTACAAAAATATATGCATTAACATGAAGC
TTTGCTGGAGTATTTGTGCAAAGGGTAAGTTATGCGAAGGTAAACAATGCTTCATTTGTTCTATCTAATCTAAATTTTAAAAG
CTTTTTGTAAGAAAAATATGGCCAGGCATTTACCAACAGGCAAGATCTAGCAGCTTACCTTATTTTTATTCCGGATGTTTAGT
CATGCATCCGAATTTCCGGCATGATAGCTCACTTCTTTCATGTGTAAATAATTCCCTCTCCAAGGGATTCCTTAATTTATTTCT
ATCCTAGCAGTTCGTACATATCTATATTTTGTGTGGAGCGTTTTCTATTTCATTTTCTTTTACTTTGTTTTGATATATGGATTCT
GACTGTAGTAATTCAGTTTTTCCACCAATTGTTGGATGCTTAAATTACCTTCAAGAAGTCTTTTTTCCCGCTCCCACATAGCAT
GCAGCAGTTATATGCATCATTTGACTGGCTGTGTCATACAGTTAAAAGGTTAGTATTTTTGCTCATACAAGGAACAGATTCAG
TTCAGTTAATTTAGCTCTATTGGTTGTATCAGTAAAACGTGGAAGTGTGCAATTTTGCTGTACATACTCCCCTATTTGTATCGG
TTCAGTTAATTCAACTCTAATTATGTTAGTTGTAAAACATGGAAGAGTTGTTCGCTGGGGTTCTTTTCCCCCCTCTCCTTCTTT
AATGAATGATATGCATACTCCGTATTCTAGAAAACAAAATACATATCCCCTATATATGCCCTTCTTATCCTTTCTTAAAAATA
ACTTGATACGTACAAGAGAATAATTACTTCCTGTAGATCTCCTTCCAAGTTTATTTACTTGATCCTTTTTCCTTTCACTTCCCTT
GACCTATTTTTTGGTGCACTGACATGGTGTCTTCAGTGGAAAATTCTGATGCTTCTATGCTTTCATGACTGAATATTTTAAATA
TGATTAAAAATATGTTATTTTCTCATGGAAAATCAAGTTATAAGTAGCTTAAATGCTTATTGCTGTTGGCATGAGATCTATGG
GTTCTCTCTTAGTAATCAAGCACGTCAACTTCTGCTTTTCAGAGAAGGTTCCATGTTGCCATGTTCTTTCTGAAGTTAAATTGG
CATTACAAATTTTGTGATCAATAACTTTCAGAAGAAAAAGGTTGCTCAAGCTACTCTTCATCAAGTTTGCACATATCATGGTA
TAGTCATCAACGACCACAATCATGATGCGCTCAAGAAAATGTTCAGGAGTTACTCAACAACACAATAGTGGAGCGGAACCC
ATCACTACTTAGTGATTTGGAAAGCAGAGTACTATTACTTTACAGAGGGAGTACATTGTGGTACTTTGATTTTTCTTTGCAAT
ATTTATTACATAGAGATAATCTAGAGCATTCTGGAGGGAAACTAATCGGTATGGAATACTGATGCATATGCATTTTCTGGTGC
AACTTGCTTCTAGGATGCGGCAACCATTTGAGAAAATTTTATACTTCCTCTGTCCTTAATTATTTGTTAGATAAATGGATGTG
ATACGATCAAGGATCCTCAAAAAGATTTATCTCCCATTTCAAGATAATTTGCCAAACCGTTGAGAGCCAGGACGTGCATCCA
GTAGCAACTTCAACATTGTCTACGCATCAAACAAACCACCTGAACAAGAGCAAAAGAACCAGCTTCATCTATCTACCACTAC
ATTGTAGAGACACACCCAGCTGGAGGATCAACATCTCAAGTCTAGGAAGAGAACAAGAGCTTCACAATTCTGCATTCAGTAT
CCACTATCACTACGTGTTGTAGATCACCTATGCATGTACCAACTAGATGTACCTTTTCCTGATCACCTATGCATGTATCAACT
ACATGTACCTATCCTTGTATGAACTATTTTGCTACAATCGAGTTTTGAAATGAC

> NewGene_945_Genomic_DNA

CATGTGTTAAAAAACCGATAAAGAATAAAAAGAAAACCAAGAAATACAAAGAAAACCAATAAAGAAACAAAAGAGAACC
AAAAGTATAACAAAGAATCTAGTGAAAAAACCAAAGAAGGTATAGCAAAGGAAAAAATAAAAACCATAGAAACCTGGGG

GAAAAAAGAAAAAAAAAAGGAAAAGAAAAACAAAGAAATCCGAGCTAGCGAGCGATTGCGCCGATGAGTTAGCCCGATTCT
CTAATTCGTTGGATGTTTCTAATAGGAATCGGAACGGGCATGACATAGTCCTGACAGCAATCAATCGAGTCCGCCACGATTC
ACCCGAACCAATCTACTGCCGCTGCCAGACGTTTTTGCAGCGGATGTCGTCGGGTTGGTTTCATGAGTACAACCGGTTGGCA
AGCCACGACACCGACGATCAGAACCTGCTGCCCCCGACCCGAGAATTACAAAAGTCTCCTTTTTTTAGGATGAATTCCTACTC
GAGATCATTCCACACCTCCAAACGCAGACGCAGACCCAAACGCCGTAGGCCCGATCCATGCAGCACAACAGTCAGCCACCT
GTTACTCAGAAGAAAAAAAGGAGCAGCCAGCCACCTGTGCATGTGTCGCCCTACCCATGGTCTTCTTTCCCCGGCCCTACAC
CCTACATAACTAGAATATTAGGCTCGTAAGGGTTTAATTTACCTGGTTTTTTTGGAGTTTTTTTAATGCCCTTGTTCGCATGAC
GCGTGTGATGGGAGTGTACCTCCTTTTTTTATCTCACTGAGCCTTTCACGTGAGATGGTAGTACGGTGGTTGCTGGTTTGACT
GAATTGTGGTGTGCCACGTACTCTCCACGTGTTCGGCTACCATGCATGAGATAATCTCTAGAAAACTCGGGTGAAGTCCTCCA
CCGCCCTATGGGCCTACAAAAAGCCCACCTCACCCCGATCAAATGAAACCCTCGCTTCTCGAACCACCTCTCCTCTACGGGTC
TCCAGGGTTGCGGAAAAAAAAAAGCGGCGCCAGCAGGCTACGGGGAGGAGGGCAGCGCCCTGCCGACGATTTATCTGGCTAC
TTCCTCCCCGTCGATCCCTGCCTGGACCATACCGTCTCCATCTTGAAGAAGGCTCGATCTTTTCCGGCGTCAGGTGAGCAGCT
GGGTGTCCTTTTTTTCCTGCGGTTTATGGCCGGCGGTGGCCTGAATCTAAGTGGTGGTTGCCGTCCGGACCGGCCGCCTTCTTT
TTGTTCTTCTCCTTGCCTTCCTTGTCACCTCTCCCAATCGCAAGTGCACGCCTCCACGAGAGCAGGAACAGAGCAGGCATTCA
TGGAGCAGATTCCTTTTTTTTCTCCACTCCTCTTGCTCCCTTCTGATCGAGCTCCGCGTAGAGGAGGGGCTCGAGCCAGGTGA
TGGCCAACACTCCGCCACGGAGGCCAACATGAGGAGGTGCCCAATCTCGCTGTTGCTCCCCTTTGCGGTGAGTGCACGCCGG
CTGCCCCTCGCACTCTCTTGGCCATGTGATATGTCGCGTGCATGTGCGGCTTCTGTGCTCCTGCTGCAGCTGTGTCATGTGTGT
GCTAGCTGATGCATTCGCGTGTCTGCCTGTAGTATATCCGCCCCGGTCCAGCTGTAGCATTCACCTTTTCCGGTATCGGTGTTC
AGGTCGACTGCAATGTGTTCAGTCAATTGACCAAACTAAGCCAACATAAAAAAGATGTGTGCTTTTTTCAGTATGCAACTAA
CATGGCATTAGCTCAACACTAGGGATGAAGTATTTACACGTTTTCTGTTCCATGGATGCACTATTTTGTGGACGTGGCTGGAG
CAATTAGTTCAGTGCATGGTTGCCTTTTTACAAGTGTTCACTTTGTTCCTGGTTTTAGTAATTATGATGCCAATATGCTTATGG
CGTGTACTTGAATATCTTTTATGATTTTTTTATGTAATTGGATGATCTCTTTCTTATTTGCAAATTAATATCACTTGACTATTGT
CTATTTGTCTTTTGTTTCAGATTGATGAGTTCGTGATGTACGAAAAGAGAATGCCTTAGAAAGACCGGTCATACGAAAGAGC
ACTTATTACTATTGAAATATTATGGTGACGATGTTTAGCAGTCATAAAAGGTATATCTTTTCTCAGATTCATATGGGCTGCCC
TTCAAAGATAAATGTAGAAACAAAGTAGGGAAGATTGTAAGATTGTCCATGGTATTACTTTAATGTGGAGAAATATCACATG
CAAATTTAGCAATCCGTAATTGTAGTTTTTTACTGGAATGTTTATTTCATAGAATTACAAATTATCATGTTACTTCTACTTCCT
TATGGTATTATACTGTGCCTTATCTCATGACCTAGGGACATTGGTGTCAAGTTTCACATACTTGAACTGTTTAATTGATTGCTT
CCATAAGATCTTCTCCTCAACGTTCCTATGTGTGATGATGTAATCCAGATCAGGCTTTGAGCTGTACTTTTTCCCTCAGCAATA
CTTTCAGTGAGAAGTAGCAACACTAATCCATCAATATAGATTATGCGAAGTATTTAACATTGAGGCTGCGTTCGTTATGCAG
GGAGAGAAAATGAAGGTTAAAAATACTTCAAAGGAATAGGACTGAAGGCTACCATAGAACCTACAGGGTCTAGGAACACA
GGAATAAGAGAAAGTAGACTGTTCGGATCACAGCAGCCTTAAGGGTTTTTAAATTGGCCACAAGGCAGCTTTGTCTCTATCT
CTCACCCCATCACGTCACACATCAGCTCATTTTAATTAGCTCTGCATGCTGACTGCCACGATCAGAGCACCTCGTCTCGGTCT
TCCGCGAAAAACATGAGCTCCGGGCAGATTTTTCAATTCCTGTGAAATCGGCATCGAAAGGACAGGTTTCCTTTGGATCACT
GTAGGTCATTCCTGCGTTTCGAACACCACAAAGGAAAATAAACCTTCATGAACAGTATTCCTTCATGATTCCTGCAAAAATCC
TATGAACCGAACGGAGCCTGAATATGGTCCAGTTAGTGGCGTATTATTCCTTGCATTCAACACGTGTGTATAACACCTCGACG
ATGTGTTATTCTATATTGAAGTTTTAATGAACCTGAACTCTTCTGATTATTTTGTGTAAGCATGAACTCTTGGCGTGCAGGTGT
AAGTTGATTTTTTTTTCAAAAAAAAGGTGCAAGTCCTATCAGGCACATGGACAACTAACATCATGCTTCCTCTGTTTAGACAT
TACTCTACCACGTAGTTGGTCTTTGGTTCTCTAGGAGATCGTGGTCCATATTTGATTAACTTACAAAAATATATGCATTAACA
TGAAGCTTTGCTGGAGTATTTGTGCAAAGGGTAAGTTATGCGAAGGTAAACAATGCTTCATTTGTTCTATCTAATCTAAATTT
TAAAAGCTTTTTGTAAGAAAAATATGGCCAGGCATTTACCAACAGGCAAGATCTAGCAGCTTACCTTATTTTTATTCCGGATG
TTTAGTCATGCATCCGAATTTCCGGCATGATAGCTCACTTCTTTCATGTGTAAATAATTCCCTCTCCAAGGGATTCCTTAATTT
ATTTCTATCCTAGCAGTTCGTACATATCTATATTTTGTGTGGAGCGTTTTCTATTTCATTTTCTTTTACTTTGTTTTGATATATG
GATTCTGACTGTAGTAATTCAGTTTTTCCACCAATTGTTGGATGCTTAAATTACCTTCAAGAAGTCTTTTTTCCCGCTCCCACA
TAGCATGCAGCAGTTATATGCATCATTTGACTGGCTGTGTCATACAGTTAAAAGGTTAGTATTTTTGCTCATACAAGGAACAG
ATTCAGTTCAGTTAATTTAGCTCTATTGGTTGTATCAGTAAAACGTGGAAGTGTGCAATTTTGCTGTACATACTCCCCTATTTG
TATCGGTTCAGTTAATTCAACTCTAATTATGTTAGTTGTAAAACATGGAAGAGTTGTTCGCTGGGGTTCTTTTCCCCCCTCTCC
TTCTTTAATGAATGATATGCATACTCCGTATTCTAGAAAACAAAATACATATCCCCTATATATGCCCTTCTTATCCTTTCTTAA
AAATAACTTGATACGTACAAGAGAATAATTACTTCCTGTAGATCTCCTTCCAAGTTTATTTACTTGATCCTTTTTCCTTTCACT
TCCCTTGACCTATTTTTTGGTGCACTGACATGGTGTCTTCAGTGGAAAATTCTGATGCTTCTATGCTTTCATGACTGAATATTT
TAAATATGATTAAAAATATGTTATTTTCTCATGGAAAATCAAGTTATAAGTAGCTTAAATGCTTATTGCTGTTGGCATGAGAT
CTATGGGTTCTCTCTTAGTAATCAAGCACGTCAACTTCTGCTTTTCAGAGAAGGTTCCATGTTGCCATGTTCTTTCTGAAGTTA
AATTGGCATTACAAATTTTGTGATCAATAACTTTCAGAAGAAAAAGGTTGCTCAAGCTACTCTTCATCAAGTTTGCACATATC
ATGGTATAGTCATCAACGACCACAATCATGATGCGCTCAAGAAAATGTTCAGGAGTTACTCAACAACACAATAGTGGAGCG
GAACCCATCACTACTTAGTGATTTGGAAAGCAGAGTACTATTACTTTACAGAGGGAGTACATTGTGGTACTTTGATTTTTCTT
TGCAATATTTATTACATAGAGATAATCTAGAGCATTCTGGAGGGAAACTAATCGGTATGGAATACTGATGCATATGCATTTTC
TGGTGCAACTTGCTTCTAGGATGCGGCAACCATTTGAGAAAATTTTATACTTCCTCTGTCCTTAATTATTTGTTAGATAAATGG
ATGTGATACGATCAAGGATCCTCAAAAAGATTTATCTCCCATTTCAAGATAATTTGCCAAACCGTTGAGAGCCAGGACGTGC
ATCCAGTAGCAACTTCAACATTGTCTACGCATCAAACAAACCACCTGAACAAGAGCAAAAGAACCAGCTTCATCTATCTACC
ACTACATTGTAGAGACACACCCAGCTGGAGGATCAACATCTCAAGTCTAGGAAGAGAACAAGAGCTTCACAATTCTGCATTC
AGTATCCACTATCACTACGTGTTGTAGATCACCTATGCATGTACCAACTAGATGTACCTTTTCCTGATCACCTATGCATGTATC
AACTACATGTACCTATCCTTGTATGAACTATTTTGCTACAATCGAGTTTTGAAATGACTTACCTAATATTTATAGAAAATCTA
AATAGCTTATCAATCTACATGGCGCGGCGTGCCGCCGCGCACTACCCGCTAGTACTAAGAATACTAGGCCCGTAAGGGTTCC
TTTTTTCTAGGTTTTTTGGAATTTTTTTACAGCCTCTTGGTAAAACTACGCGTGTAACTATTGAATAGAGAGTCCCTACGATGC
CGGATGGTCGTTCAGATTCTTTTAGCAAACAGTTTAATCACGTGGCCTATGGAACTGCCTAGATATTTGCATGGATTTTGGAA
GGCGGTAGAACCAGACAGCGATCTGG

>TAES_LNC009446.1: long non-coding RNA

CTTGAATATCTTTTATGATTTTTTTATGTAATTGGATGATCTCTTTCTTATTTGCAAATTAATATCACTTGACTATTGTCTATTT
GTCTTTTGTTTCAGATTGATGAGTTCGTGATGTACGAAAAGAGAATGCCTTAGAAAGACCGGTCATACGAAAGAGCACTTAT
TACTATTGAAATATTATGGTGACGATGTTTAGCAGTCATAAAAGGTGTAAGTTGATTTTTTTTTCAAAAAAAAGGTGCAAGTC
CTATCAGGCACATGGACAACTAACATCATGCTTCCTCTGTTTAGACATTACTCTACCACGTAGTTGGTCTTTGGTTCTCTAGG
AGATCGTGGTCCATATTTGATTAACTTACAAAAATATATGCATTAACATGAAGCTTTGCTGGAGTATTTGTGCAAAGGGTAA
GTTATGCGAAGGTAAACAATGCTTCATTTGTTCTATCTAATCTAAATTTTAAAAGCTTTTTGTAAGAAAAATATGGCCAGGCA
TTTACCAACAGGCAAGATCTAGCAGCTTACCTTATTTTTATTCCGGATGTTTAGTCATGCATCCGAATTTCCGGCATGATAGC
TCACTTCTTTCATGTGTAAATAATTCCCTCTCCAAGGGATTCCTTAATTTATTTCTATCCTAGCAGTTCGTACATATCTATATTT
TGTGTGGAGCGTTTTCTATTTCATTTTCTTTTACTTTGTTTTGATATATGGATTCTGACTGTAGTAATTCAGTTTTTCCACCAAT
TGTTGGATGCTTAAATTACCTTCAAGAAGTCTTTTTTCCCGCTCCCACATAGCATGCAGCAGTTATATGCATCATTTGACTGGC
TGTGTCATACAGTTAAAAGGTTAGTATTTTTGCTCATACAAGGAACAGATTCAGTTCAGTTAATTTAGCTCTATTGGTTGTAT
CAGTAAAACGTGGAAGTGTGCAATTTTGCTGTACATACTCCCCTATTTGTATCGGTTCAGTTAATTCAACTCTAATTATGTTA
GTTGTAAAACATGGAAGAGTTGTTCGCTGGGGTTCTTTTCCCCCCTCTCCTTCTTTAATGAATGATATGCATACTCCGTATTCT
AGAAAACAAAATACATATCCCCTATATATGCCCTTCTTATCCTTTCTTAAAAATAACTTGATACGTACAAGAGAATAATTACT
TCCTGTAGATCTCCTTCCAAGTTTATTTACTTGATCCTTTTTCCTTTCACTTCCCTTGACCTATTTTTTGGTGCACTGACATGGT
GTCTTCAGTGGAAAATTCTGATGCTTCTATGCTTTCATGACTGAATATTTTAAATATGATTAAAAATATGTTATTTTCTCATGG
AAAATCAAGTTATAAGTAGCTTAAATGCTTATTGCTGTTGGCATGAGATCTATGGGTTCTCTCTTAGTAATCAAGCACGTCAA
CTTCTGCTTTTCAGAGAAGGTTCCATGTTGCCATGTTCTTTCTGAAGTTAAATTGGCATTACAAATTTTGTGATCAATAACTTT
CAGAAGAAAAAGGTTGCTCAAGCTACTCTTCATCAAGTTTGCACATATCATGGTATAGTCATCAACGACCACAATCATGATG
CGCTCAAGAAAATGTTCAGGAGTTACTCAACAACACAATAGTGGAGCGGAACCCATCACTACTTAGTGATTTGGAAAGCAG
AGTACTATTACTTTACAGAGGGAGTACATTGTGATAAATGGATGTGATACGATCAAGGATCCTCAAAAAGATTTATCTCCCA
TTTCAAGATAATTTGCCAAACCGTTGAGAGCCAGGACGTGCATCCAGTAGCAACTTCAACATTGTCTACGCATCAAACAAAC
CACCTGAACAAGAGCAAAAGAACCAGCTTCATCTATCTACCACTACATTGTAGAGACACACCCAGCTGGAGGATCAACATCT
CAAGTCTAGGAAGAGAACAAGAGCTTCACAATTCTGCATTCAGTATCCACTATCACTACGTGTTGTAGATCACCTATGCATGT
ACCAACTAGATGTACCTTTTCCTGATCACCTATGCATGTATCAACTACATGTACCTATCCTTGTATGAACTATTTTGCTACAAT
CGAGTTTTGAAATGACTTACCTAATATTTATAGAAAATCTAAATAGCTTATCA
