# Supplementary material for: TaMIR397-6A and -6B Homoeologs Encode Active miR397 Contributing to the Regulation of Grain Size in Hexaploid Wheat
Source: Int J Mol Sci. 2024 Jul 13;25(14):7696. doi: 10.3390/ijms25147696 (PMC11276883; doi:10.3390/ijms25147696)
Supplement: Supplementary file 1 [file ijms-25-07696-s001.zip › Supplementary file S3.pdf]

Supplementary File S3:
The expression of three TamiR397a-regulated genes in developing wheat grains

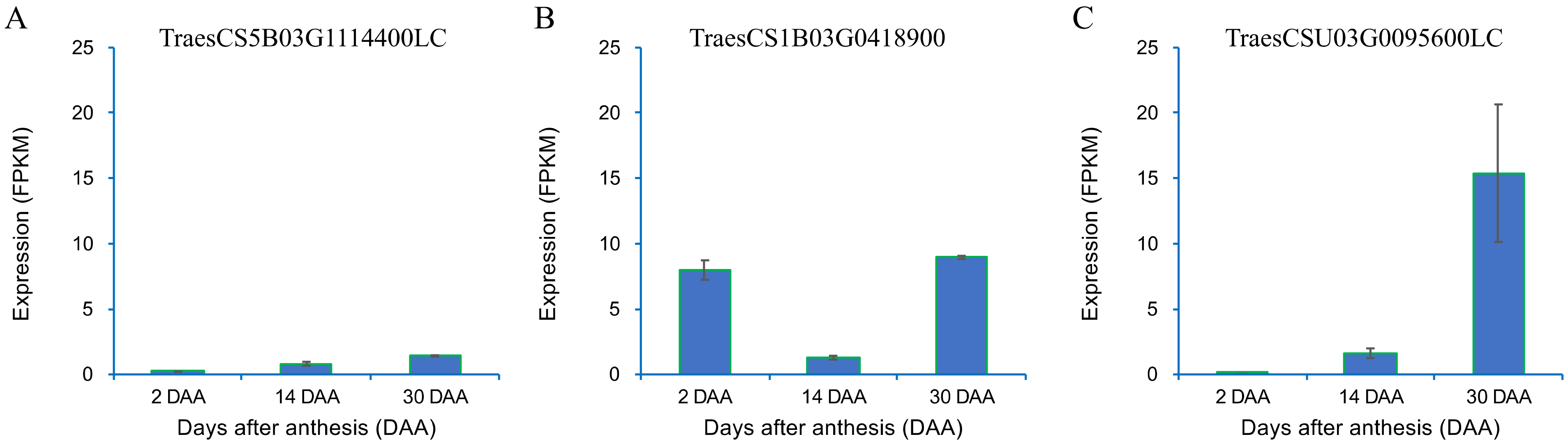

The expression analysis is conducted on WheatExp (https://wheat.pw.usda.gov/WheatExp)
