# Supplementary material for: TaMIR397-6A and -6B Homoeologs Encode Active miR397 Contributing to the Regulation of Grain Size in Hexaploid Wheat
Source: Int J Mol Sci. 2024 Jul 13;25(14):7696. doi: 10.3390/ijms25147696 (PMC11276883; doi:10.3390/ijms25147696)
Supplement: Supplementary file 1 [file ijms-25-07696-s001.zip › Supplementary file S4.pdf]

Supplementary File S4: The PCR profile

A. Gene Cloning of TaMIR397a

Composition of PCR reaction

Serial Number	Constituents	Amount
1	Primer: P1674	1 µL
2	Primer: P1675	1 µL
3	LA Taq	0.2 µL
4	10×LA Taq Buffer II	2 µL
5	dNTP mixture	1.8 µL
6	Template cDNA	1 µL
7	ddH_2O	Up to 20 µL

Conditions of PCR amplification

Serial Number	Phases	Temperature	Time	Cycles
1	Initial temperature	95 °C	5 minutes	
2	Denaturation	95 °C	30 seconds	30
3	Annealing	60 °C	30 seconds	
4	Extension	72 °C	30 seconds	
5	Final extension	72 °C	5 minutes	
6	Hold	4 °C	30 minutes	

B. Identified of recombinant *E. coli* colonies by PCR

Composition of PCR reaction

Serial Number	Constituents	Amount
1	Primer: P0085 or specific primer	1 µL
2	Primer: P0086 or specific primer	1 µL
3	2×Taq Plus Master Mix	10 µL
4	Recombinant *E. coli* colonies	1 µL
5	ddH_2O	Up to 20 µL

Conditions of PCR amplification

Serial Number	Phases	Temperature	Time	Cycles
1	Initial temperature	95 °C	5 minutes	
2	Denaturation	95 °C	30 seconds	30
3	Annealing	60 °C	30 seconds	
4	Extension	72 °C	1 minutes	
5	Final extension	72 °C	5 minutes	
6	Hold	4 °C	30 minutes	

C. Cloning of TaMIR397a-6A or TaMIR397a-6B fragment for functional validation

Composition of PCR reaction

Serial Number	Constituents	Amount
1	Primer: P2141	1 µL
2	Primer: P2142	1 µL
3	LA Taq	0.2 µL
4	10×LA Taq Buffer II	2 µL
5	dNTP mixture	1.8 µL
6	Specific gene harbored vector	0.1 µL
7	ddH_2O	Up to 20 µL

Conditions of PCR amplification

Serial Number	Phases	Temperature	Time	Cycles
1	Initial temperature	95 °C	5 minutes	
2	Denaturation	95 °C	30 seconds	30
3	Annealing	60 °C	30 seconds	
4	Extension	72 °C	30 seconds	
5	Final extension	72 °C	5 minutes	
6	Hold	4 °C	30 minutes	

D. Cloning of the *Lac10* gene through PCR amplification

Composition of PCR reaction

Serial Number	Constituents	Amount
1	Primer: LPF	1 µL
2	Primer: LPR	1 µL
3	LA Taq	0.2 µL
4	10×LA Taq Buffer II	2 µL
5	dNTP mixture	1.8 µL
6	Template cDNA	1 µL
7	ddH_2O	Up to 20 µL

Conditions of PCR amplification

Serial Number	Phases	Temperature	Time	Cycles
1	Initial temperature	95 °C	5 minutes	
2	Denaturation	95 °C	30 seconds	30
3	Annealing	60 °C	30 seconds	
4	Extension	72 °C	30 seconds	
5	Final extension	72 °C	5 minutes	
6	Hold	4 °C	30 minutes	

E. Cloning of the *Lac10m* fragment through PCR amplification

Composition of PCR reaction

Serial Number	Constituents	Amount
1	Primer: LPF or P2329	1 µL
2	Primer: LPR or P2330	1 µL
3	LA Taq	0.2 µL
4	10×LA Taq Buffer II	2 µL
5	dNTP mixture	1.8 µL
6	Template cDNA	1 µL
7	ddH_2O	Up to 20 µL

Conditions of PCR amplification

Serial Number	Phases	Temperature	Time	Cycles
1	Initial temperature	95 °C	5 minutes	
2	Denaturation	95 °C	30 seconds	30
3	Annealing	60 °C	30 seconds	
4	Extension	72 °C	30 seconds	
5	Final extension	72 °C	5 minutes	
6	Hold	4 °C	30 minutes	

F. Expression analysis through qRT-PCR

Composition of PCR reaction

Serial Number	Constituents	Amount
1	SYBR Green I PCR Master Mix	10 µL
2	10 µM specific primer pairs (F/R)	1 µL / 1 µL
3	cDNA	2 µL
4	Nuclease-free water	6 µL

Conditions of PCR amplification

Serial Number	Phases	Temperature	Time	Cycles
1	Initial temperature	95 °C	3 minutes	
2	Denaturation	95 °C	10 sec	40 cycles
3	Annealing and extension	60 °C	30 sec	
4	Ramp slowly from 60o to 95 °C to generate a dissociation curve			
