# Supplementary material for: TaMIR397-6A and -6B Homoeologs Encode Active miR397 Contributing to the Regulation of Grain Size in Hexaploid Wheat
Source: Int J Mol Sci. 2024 Jul 13;25(14):7696. doi: 10.3390/ijms25147696 (PMC11276883; doi:10.3390/ijms25147696)
Supplement: Supplementary file 1 [file ijms-25-07696-s001.zip › Supplementary Table S1.pdf]

Supplementary Table S1. Primers used in this study

Primer No.	Sequence (5´-3´)	Function
P1674	AGCCAGATGCAACGAGATACTAC	*TaMIR397a* cloning (F)
P1675	CGCGAAGCTGCGAAAGGC	*TaMIR397a* cloning (R)
P0085	ATCGGTGCGGGCCTCTT	T-vector common primer (F)
P0086	GGCACCCCAGGCTTTACAC	T-vector common primer (R)
P1707	GCCTGTGTCAATGGTGTCGGG	*TaMIR397a-6A* specific primer (R)
P1706	GCGTTGATGAACCGTCCGG	*TaMIR397a-6B* specific primer (F)
P1708	CGGCACATGAGCATACCTGATG	*TaMIR397a-6D* specific primer (R)
P2141	ATACTCGAGACTGGGGAAGACGAAGAGG	*TaMIR397a-6A* or *-6B* cloning for functional identification (F)
P2142	ATAGGGCCCAGCTCCTCTTCTCCGCTTCAT	*TaMIR397a-6A* or *-6B* cloning for functional identification (R)
LPF	TCGCCATGGGCGAGTGGTTTAACACG	*Lac10* cloning primer (F)
LPR	ATTAGATCTACGGTGGTGTTGTCGTAGG	*Lac10* cloning primer (R)
P2329	TCTGCTCCGGCTGATCAACGCATCTGCGCTCAACGACGAGCTT	*Lac10* mutation primer (F)
P2330	AAGCTCGTCGTTGAGCGCAGATGCGTTGATCAGCCGGAGCAGA	*Lac10* mutation primer (R)
P0929	TTGAGTGCAGCGTTGATGAAC	qRT-PCR for miR397a
P1303	CCTTCGGGGACATCCGATAAA	qRT-PCR for U6 (F)
P1304	CCTTCGGGGACATCCGATAAA	qRT-PCR for U6 (R)
P3184	CAGGACATCCCTTGCTGCAATC	qRT-PCR for *TPR* (F)
P3188	CAACCGCAGCCTTCGCCA	qRT-PCR for *TPR* (R)
P3192	AGATGTTCTGACAGCGACATGGAC	qRT-PCR for *F-box/LRR* (F)
P3194	AAGGGTCTCGCTCGATCAC	qRT-PCR for *F-box/LRR* (R)
P3198	CGCGGCACGGACGACATC	qRT-PCR for *STP* (F)
P3200	GGTCACGATGGACACGAAGG	qRT-PCR for *STP* (R)
P3204	TGGAGCGGAACCCATCACTAC	qRT-PCR for *NG 945* (F)
P3205	CCTGGCTCTCAACGGTTTGG	qRT-PCR for *NG 945* (R)
P2006	GCGAAGCCAGCAACCTATGATC	qRT-PCR for *GADPH* (F)
P2008	AAGGCTTTGAACACTCCTCTGG	qRT-PCR for *GADPH* (R)
P1715	GGAGCTCGGGTACCCCTACTACTGGGGAAGACGAAGAGG	*TaMIR397a* overexpression (F)
P1716	GAAAGCTCGATCCCCAGCTCCTCTTCTCCGCTTCATG	*TaMIR397a* overexpression(R)
P1693	CGCGGTGGAGCTCGGGTTCATCAACGCTACTGCACTCAAGTTGTTGTTGTTATGG	*STTM-miR397a* fragment amplification (F)
P1694	AAGCTCGATCCCCGGTTGAGTGCAGTAGCGTTGATGAACATTCTTCTTCTTTAGACCA	*STTM-miR397a* fragment amplification (R)
P0745	GTTGTTGTTGTTATGGTCTAATTTAAATATGGTCTAAAGAAGAAGAAT	*STTM-miR397a* fragment amplification templet
P1635	GCTCACCCTGTTGTTTGGTGTTAC	Transgenic identification (F)
P1696	ACTTCGGTCATTAGAGGCCACG	Transgenic identification (R)
P2009	GGAGCGCACACACACACAAC	Expression detection of transgene (F)
P1677	AGCTCCTCTTCTCCGCTTCATG	Expression detection of transgene *TaMIR397a* (R)
P1733	AGCTCGATCCCCGGTTGAG	Expression detection of transgene *STTM-miR397a* (R)
